# Comprehensive Analysis Uncovers Prognostic and Immunogenic Characteristics of Cellular Senescence for Lung Adenocarcinoma

**DOI:** 10.3389/fcell.2021.780461

**Published:** 2021-11-16

**Authors:** Weihao Lin, Xin Wang, Zhen Wang, Fei Shao, Yannan Yang, Zheng Cao, Xiaoli Feng, Yibo Gao, Jie He

**Affiliations:** ^1^ Department of Thoracic Surgery, National Cancer Center/National Clinical Research Center for Cancer/Cancer Hospital, Chinese Academy of Medical Sciences and Peking Union Medical College, Beijing, China; ^2^ Department of Pathology, National Cancer Center/National Clinical Research Center for Cancer/Cancer Hospital, Chinese Academy of Medical Sciences and Peking Union Medical College, Beijing, China; ^3^ State Key Laboratory of Molecular Oncology, National Cancer Center/National Clinical Research Center for Cancer/Cancer Hospital, Chinese Academy of Medical Sciences and Peking Union Medical College, Beijing, China

**Keywords:** lung adenocarcinoma, cellular senescence, senescence-associated secretory phenotype, tumor microenvironment, prognosis, immunotherapy

## Abstract

Cellular senescence plays a crucial role in tumorigenesis, development and immune modulation in cancers. However, to date, a robust and reliable cellular senescence-related signature and its value in clinical outcomes and immunotherapy response remain unexplored in lung adenocarcinoma (LUAD) patients. Through exploring the expression profiles of 278 cellular senescence-related genes in 936 LUAD patients, a cellular senescence-related signature (SRS) was constructed and validated as an independent prognostic predictor for LUAD patients. Notably, patients with high SRS scores exhibited upregulation of senescence-associated secretory phenotype (SASP) and an immunosuppressive phenotype. Further analysis showed that SRS combined with immune checkpoint expression or TMB served as a good predictor for patients’ clinical outcomes, and patients with low SRS scores might benefit from immunotherapy. Collectively, our findings demonstrated that SRS involved in the regulation of the tumor immune microenvironment through SASP was a robust biomarker for the immunotherapeutic response and prognosis in LUAD.

## Introduction

Lung cancer has the highest incidence and mortality of cancer worldwide (Sung, et al., 2021). The 5-years survival rate is less than 20% (Miller, et al., 2019). Lung adenocarcinoma (LUAD) is the main histological subtype of non-small-cell lung cancer (NSCLC), accounting for approximately 60% of NSCLC cases (Behera, et al., 2016). Although understanding LAUD genomics and breakthroughs of targeted therapies and immunotherapies have substantially expanded treatment modalities, challenges associated with LAUD remain elusive. Therefore, better prognostic tools and biomarkers accurately predicting the characteristics of tumors are urgently needed to stratify patients and personalize treatment strategies for LUAD.

Cellular senescence is one of the key processes of ageing (Campisi 2013) and serves as a link between ageing and cancer (Partridge, et al., 2018). However, the linkage of senescence and cancer, especially in lung cancer, is complex and poorly understood at present. Previous studies have highlighted that the existence of senescence plays a double-edged sword in the process of tumorigenesis and development. On the one hand, in the context of senescent cells entering permanent cell cycle arrest, senescence ensures tissue homeostasis and prevents tumorigenesis (Krizhanovsky, et al., 2008; Perez-Mancera, et al., 2014). Senescence acts as a barrier from tumor development in early tumorigenesis when it is followed by immune clearance and tissue remodelling (Xue, et al., 2007). On the other hand, cellular senescence can present a detrimental outcome when senescent cells are not cleared by the immune system and accumulate. This accumulation promotes the senescence-associated secretory phenotype (SASP), which releases cytokines, growth factors, extracellular matrix (ECM) components and ECM-degrading enzymes (Lasry and Ben-Neriah 2015; Lopes-Paciencia, et al., 2019), leading to both the ageing process and tumor development (Coppe, et al., 2010; Cuollo, et al., 2020). Therefore, an improved understanding of the impact of senescence on tumor immunity associated with invasion and development is required to frame novel treatment paradigms for tumors.

**FIGURE 1:**
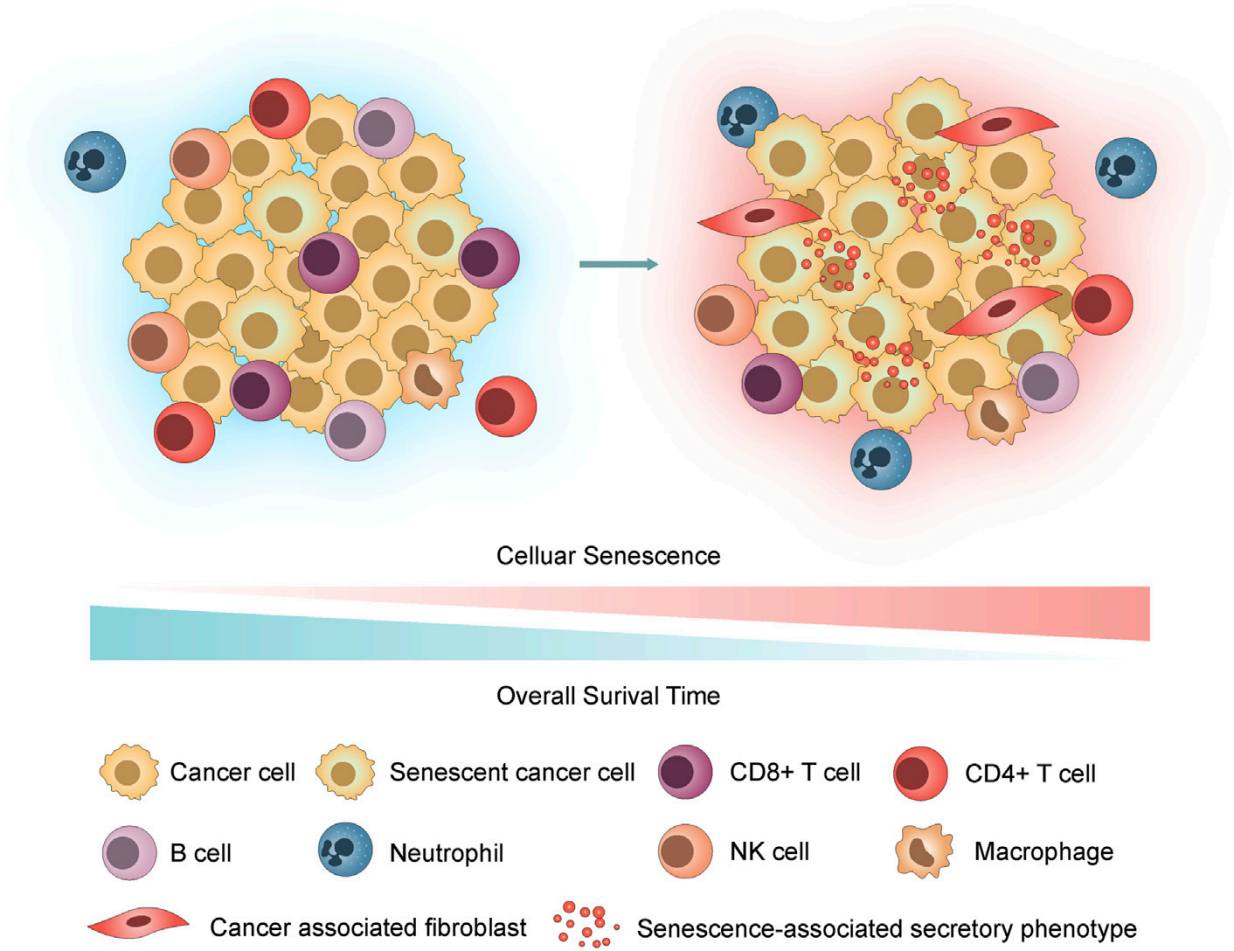
Hypothesis graph. With the accumulation of senescent cells, the inflammatory SASP remodels the TIME by recruiting immunosuppressive cells, thus promoting tumor cell invasion through immunosurveillance, proliferation, and metastasis and contributing to poor prognosis in LUAD.

According to recent studies, tumor cells can undergo senescence as an evolutionary process, including both tumor-intrinsic characteristics (dramatic gene expression changes along with chromatin remodelling and engagement of a persistent DDR) and extrinsic immune pressure (a temporal cascade in the development of SASP) (Lasry and Ben-Neriah 2015; Berben, et al., 2021; Eggert, et al., 2016; Hernandez-Segura, et al., 2018; Kumari and Jat 2021). Notably, the deleterious effects of SASP overshadow its beneficial properties (Cuollo, et al., 2020). We hypothesized that accompanied by the accumulation of senescent cells, inflammatory SASP remodels the tumor immune microenvironment (TIME) by recruitment of immunosuppressive protumorigenic cells, such as cancer-associated fibroblasts (CAFs), macrophages and neutrophils, and a decrease in cytotoxic lymphocytes (T and NK cells) and promotes tumor cell evasion from immunosurveillance, growth, and metastasis, contributing to poor prognosis in LUAD (Figure 1).

To systematically assess the correlations between cellular senescence and prognosis in LUAD, we established a novel risk model based on cellular senescence-related genes and explored their potential importance as predictive biomarkers for prognosis and immunotherapy response. Subsequently, the relationships among risk subgroups, immune checkpoints, and immune cell infiltration were thoroughly analysed based on cell senescence-related signature. Further exploration of the mechanisms suggested that tumor cellular senescence affected the TIME through SASP. This study provided new insights into the regulatory mechanisms of cellular senescence associated with the TIME and strategies for LUAD immunotherapy.

## Materials and Methods

### Data Acquisition and Processing

Clinical information and transcriptional profiles of patients with LUAD were obtained from The Cancer Genome Atlas (TCGA, https://portal.gdc.cancer.gov) and the Gene Expression Omnibus (GEO, http://www.ncbi.nlm.nih.gov/geo). After filtering, a total of 500 patients with both mRNA expression and corresponding clinical data in the TCGA cohort were included in the training cohort. Fragments per kilobase million (FPKM) data of the TCGA cohort were then transformed into transcripts per million (TPM) data for further analysis. Three additional independent datasets, GSE30219 (Rousseaux, et al., 2013) (*n* = 83), GSE31210 (Okayama, et al., 2012) (*n* = 226) and GSE50081 (Der, et al., 2014) (*n* = 127), were enrolled as the validation cohorts. For microarray data processing, the mean expression values were used when genes matched with multiple probes. Moreover, IMvigor210 (Mariathasan, et al., 2018), an immunotherapy cohort with 348 metastatic urothelial cancer patients treated with anti-PD-L1 agent, was downloaded from http://research-pub.gene.com/IMvigor210CoreBiologies/, and data processing methods were also provided in the IMvigor210CoreBiologies package. Detailed clinical information of the five patient datasets is shown in Supplementary Table S1. The flow diagram of this study is depicted in Supplementary Figure S1.

### Development and Validation of the Cellular Senescence-Related Signature

The list of genes was obtained from CellAge (Avelar, et al., 2020) (https://genomics.senescence.info/cells/), which contains manually curated data of human genes associated with cellular senescence. A total of 278 genes (Supplementary Table S2) were included in this study. We first screened cellular senescence-related differentially expressed genes (DEGs) between normal samples (*n* = 59) and tumor samples (*n* = 513) based on the thresholds of an adjusted *p* < 0.01 and | log2 (fold change) | > 1. Univariate Cox proportional hazard regression analysis was performed to identify cellular senescence-related prognostic genes (*p* < 0.001). Next, the DEGs and prognostic genes were investigated using the R package “veen” to acquire prognostic cellular senescence-related DEGs, and correlations were visualized by the R package “circlize” (Gu, et al., 2014). Least absolute shrinkage and selection operator (LASSO) Cox regression (Tibshirani 1997) was conducted with a random seed using the R package “glmnet” (Friedman, et al., 2010) to construct the risk score model (cellular senescence-related signature, SRS) for best predicting survival in the training cohort and was repeated 1,000 times. The optimal values of the penalty parameter lambda were determined through 10-fold cross-validations. Based on the median risk score calculated by SRS, patients in the training and validation cohorts were divided into high- and low-risk groups, and the performance of SRS was subsequently evaluated.

### Signature Genes Analyses

Expression of the five signature genes was analysed in The Gene Expression Profiling Interactive Analysis (GEPIA2 (Tang, et al., 2019), http://gepia2.cancer-pku.cn/) database, and these analyses were based on tumor and normal samples from the TCGA and GTEx databases. Pancancer expression analysis of the five genes was also performed using the Oncomine (https://www.oncomine.org/) database. UALCAN (Chandrashekar, et al., 2017) (http://ualcan.path.uab.edu), another powerful interactive online tool, was used to reveal the promoter methylation levels of signature genes.

### Pathway and Functional Enrichment Analysis

Kyoto Encyclopedia of Genes and Genomes (KEGG) (Kanehisa, et al., 2016) and Gene Ontology (GO) (The Gene Ontology 2019) enrichment analyses were applied using the R package clusterProfiler (Yu, et al., 2012). The DEGs between the high- and low-risk groups were subjected to pathway and functional enrichment analysis. Gene set enrichment analysis (GSEA) (Subramanian, et al., 2005) was also performed in the javaGSEA desktop application (GSEA 4.1.0) to identify the underlying pathways or processes in patients with high or low scores. Significantly enriched gene sets were defined as gene sets with a normalized enrichment score (NES) > 1.5 and *p* < 0.05.

### Correlation Between Immune Cell Infiltration and SRS

We integrated several computational tools to estimate immune cell infiltration in TCGA RNA-seq cohorts. Immune infiltration estimations for characterizing the cell composition of complex tissues from the gene expression profiles were performed using TIMER (Li, et al., 2016), EPIC(Racle, et al., 2017), xCELL (Aran, et al., 2017), CIBERSORT(Newman, et al., 2015) and quanTIseq (Finotello, et al., 2019) algorithms in TIMER2.0 (Li, et al., 2016; Li, et al., 2017; Li, et al., 2020; Sturm, et al., 2019) (http://timer.comp-genomics.org/). Using the gsva (Hanzelmann, et al., 2013) algorithm, gene signatures of tumor-infiltrating lymphocytes downloaded in TISIDB(Ru, et al., 2019) (http://cis.hku.hk/TISIDB/index.php) were also employed to estimate the immune cell infiltration level of each sample. Pearson correlation analysis was conducted to clarify the correlation between SRS and immune cell infiltration.

### Assessment of SRS and Response to Immune Checkpoint Inhibitors

The immunophenoscore (IPS), which has been demonstrated to predict patients’ response to immune checkpoint inhibitor (ICI) treatment, was downloaded from The Cancer Immunome Atlas (TCIA (Charoentong, et al., 2017), https://tcia.at). A higher IPS score indicates a better immunotherapy response. Tumor Immune Dysfunction and Exclusion (TIDE (Fu, et al., 2020; Jiang, et al., 2018), http://tide.dfci.harvard.edu/), which was developed to assess immune evasion mechanisms, is another robust biomarker used to predict immunotherapy response. A higher TIDE score means that tumor cells are more prone to escape from immunosurveillance, suggesting a lower response rate to immunotherapy. TIDE scores were obtained after unloading the input data as described in the instructions. The TMB for each patient in the TCGA cohort was calculated as the number of nonsynonymous mutations per mega-base. PD-L1 expression on tumor-infiltrating immune cells (ICs) of patients in the IMvigor210 cohort was assessed by immunohistochemistry. IC0 and IC1 exhibit low PD-L1 expression, while IC2 indicates high PD-L1 expression in our study.

### Clinical Specimens

We retrospectively collected 74 paraffin-embedded LUAD specimens and 74 adjacent normal tissues from the biobank of National Cancer Center/National Clinical Research Center for Cancer/Cancer Hospital in Chinese Academy of Medical Sciences and Peking Union Medical College (Beijing, China) and constructed tissue microarray (TMA). All the biospecimens were obtained from LAUD patients who underwent radical resection and had received no prior chemotherapy or radiotherapy. Informed consent was obtained from all patients. This study was approved by the Ethics and Research Committees of the National Cancer Center/Cancer Hospital, Chinese Academy of Medical Sciences, and Peking Union Medical College.

### Immunohistochemistry

TMA slides were incubated at 4°C overnight with primary antibodies against FOXM1 (Proteintech, 13147-1-AP, 1: 200), HJURP (Proteintech, 15283-1-AP, 1: 200), and PTTG1 (ABclonal, A8307, 1: 500) after deparaffinization, rehydration and antigen retrieval. Next, the slides were incubated with anti-rabbit secondary antibody and followed by DAB staining and hematoxylin counterstaining. Two pathologists who were blind to the information of patients independently evaluated the IHC results. The TMA sections were scored according to the percentage of positive cells and staining intensity. Staining intensity was scored as 0 (negative), 1 (weak), 2 (moderate) or 3 (strong) and the expression proportion of positive cells was scored as 1 (0–25%), 2 (26–50%), 3 (51–75%) or 4 (76–100%). The proportion and intensity scores were then integrated to obtain a final score.

### Statistical Analysis

Data analysis and graph generation were all performed in R version 3.5.1 (https://www.r-project.org), SPSS Statistics V25.0 and GraphPad Prism 8.0. For comparisons of two groups, unpaired Student’s t-test was applied to analyse the statistical significance of normally distributed variables, and the Wilcoxon rank-sum test was adopted to estimate nonnormally distributed variables. Categorical variables were compared using the $\chi^{2}$ test. The Kaplan-Meier survival curve for overall survival (OS) analysis was plotted with the R package “survminer”. Receiver operating characteristic (ROC) curves for 1-, 3-, and 5-years survival were delineated to evaluate the predictive efficacy of the SRS score, which was generated using timeROC(Blanche, et al., 2013). Univariate and multivariate Cox regression analyses were utilized to evaluate the association between OS and clinicopathological characteristics as well as SRS scores. All statistical analyses were two-tailed, and *p* < 0.05 was considered statistically significant.

## Results

### Identification of Differentially Expressed Senescence-Related Genes in LUAD

**FIGURE 2:**
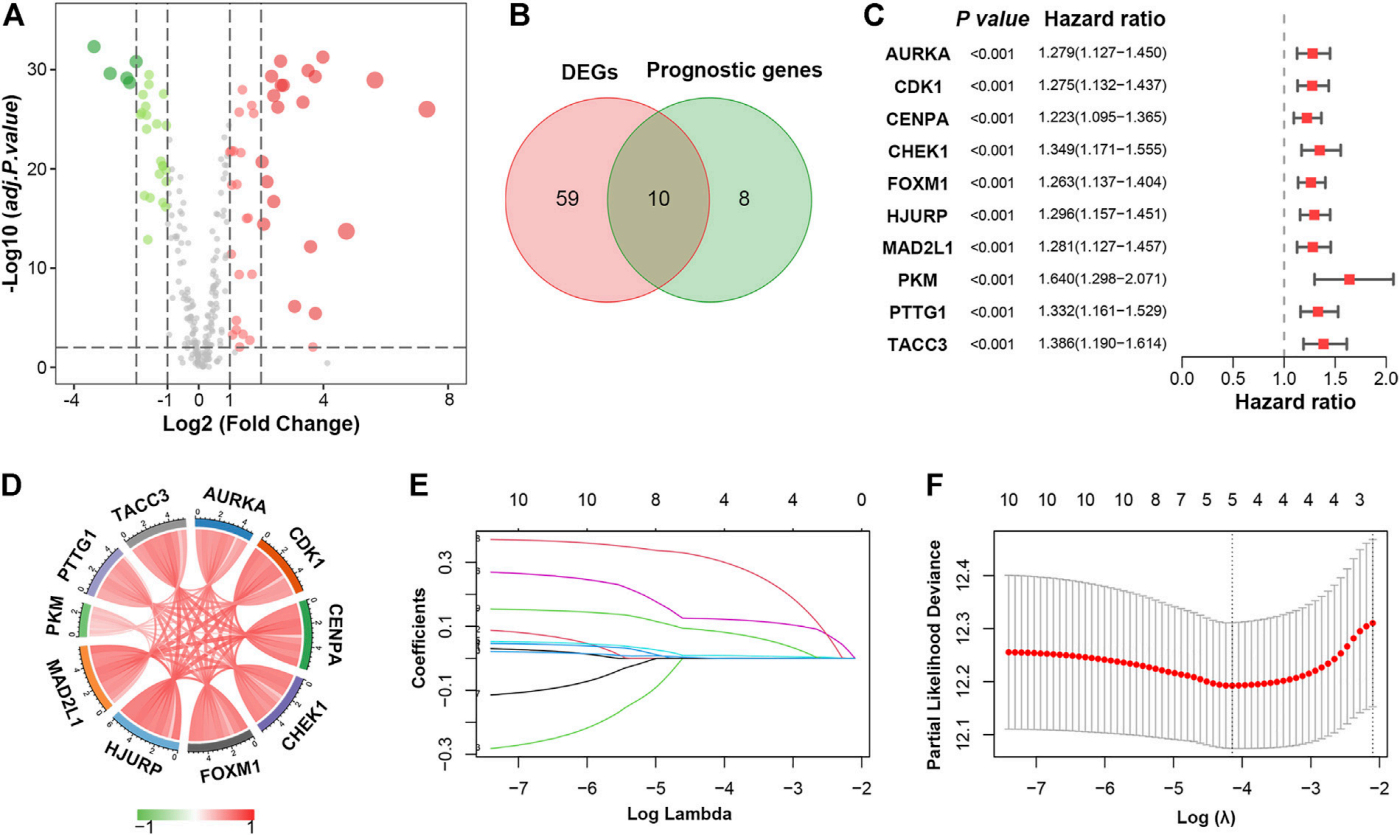
Identification of prognostic cellular senescence-related DEGs. **(A)**. Volcano plot of cellular senescence-related genes in TCGA database; red and green nodes indicate upregulated and downregulated genes, respectively. **(B)**. Venn diagram showing 10 overlapping genes in DEGs and prognostic genes. **(C)**. Forrest plot of the univariate Cox analysis of 10 overlapping genes. **(D)**. Correlation network of 10 candidate genes. **(E)**. LASSO coefficient profiles of 10 candidate genes. **(F)**. Cross-validation for tuning parameter selection in the LASSO regression.

To comprehensively characterize the expression pattern of cellular senescence-related genes, the 278 genes downloaded in CellAge (Avelar, et al., 2020) were compared in tumor tissues versus normal tissues in the TCGA-LUAD cohort, and we identified 69 differentially expressed genes (DEGs). Among them, 42 genes were upregulated, whereas 27 were downregulated (Figure 2A; Supplementary Table S3). GO and KEGG analyses were performed to clarify the biological process of the DEGs. As expected, the DEGs were remarkably enriched in cell cycle- and cellular senescence-related pathways, as they were obtained from a website of genes related to cellular senescence (Supplementary Figure S2; Supplementary Table S4).

Next, univariate Cox proportional hazard regression analysis was initially performed to identify cellular senescence-related genes associated with overall survival (OS). A total of 18 genes were significantly associated with OS (*p* < 0.001, Supplementary Table S5), ten of which overlapped with the DEGs (Figure 2B). All ten genes (AURKA, CDK1, CENPA, CHEK1, FOXM1, HJURP, MAD2L1, PKM, PTTG1 and TACC3) were upregulated in LUAD and considered risk factors (*p* < 0.001, HR > 1) (Figure 2C). Besides, 10 prognostic DEGs were positively correlated with each other (Figure 2D).

### Development of a Cellular Senescence-Related Signature in LUAD

To construct a cellular senescence-related signature (SRS) for survival prediction, the 10 genes mentioned above were analysed by LASSO-Cox regression analysis. A 5-gene signature was constructed according to the optimum λ value (Figures 2E,F). We then established a risk score formula based on the expression of the five genes for patients with LUAD: risk score = (0.0089 × expression value of FOXM1) + (0.1233 × expression value of HJURP) + (0.3092 × expression value of PKM) + (0.0851 × expression value of PTTG1) + (0.0003 × expression value of TACC3). The risk score of every patient was then calculated using this formula, and patients in the TCGA cohort were stratified into low- and high-risk groups according to the median value of the risk score.

**FIGURE 3:**
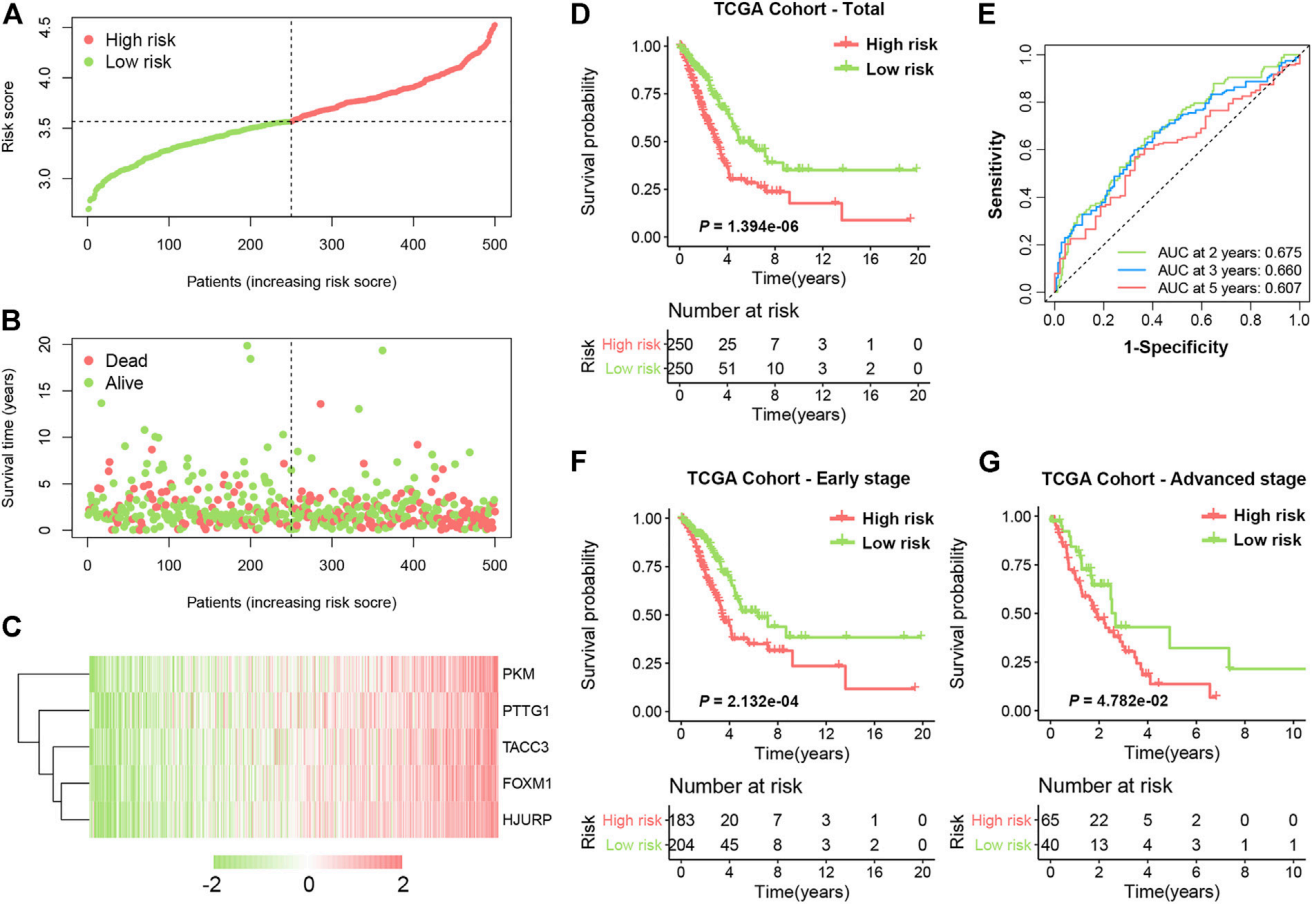
Development of SRS in TCGA cohort. **(A)**. The risk score distribution of LUAD patients in the training cohort. **(B)**. Survival status scatter plots for LUAD patients in the training cohort. **(C)**. Heatmap of the expression profiles of five signature genes in the high- and low-risk groups. **(D)**. Kaplan-Meier curves of OS in total LUAD patients of TCGA cohort based on risk score. **(E)**. Time-dependent ROC curve analysis of the prognostic model (2, 3, and 5 years). **(F)** and **(G)**. Kaplan-Meier curves of OS in patients with early-stage and advanced-stage LUAD based on risk score.

The distribution of the SRS score, the survival status, and a heatmap exhibiting the expression profiles of the selected genes in the high- and low-risk groups are presented in Figures 3A–C. Kaplan-Meier survival analysis demonstrated that patients in the high-risk group had a significantly shorter OS time compared with those in the low-risk group (Figure 3D, HR = 2.048, 95% CI 1.529–2.743, log-rank *p* < 0.0001). The 5-years survival rate of the high-risk group was 30.2%, which was significantly lower than that of the low-risk group (49.7%). Time-dependent receiver operating characteristic (ROC) analysis was performed, and the areas under the curve (AUCs) for 2-, 3-, and 5-years OS were 0.675, 0.660, and 0.607, respectively (Figure 3E). In addition, our formula also worked well when applied to patients with different clinical stages. As shown in Figures 3F,G significant difference in OS time was observed in both early-stage (HR = 1.955, 95% CI 1.357–2.816, log-rank *p* = 0.0002) and advanced-stage LUAD (HR = 1.725, 95% CI 1.034–2.879, log-rank *p* = 0.0478).

**FIGURE 4:**
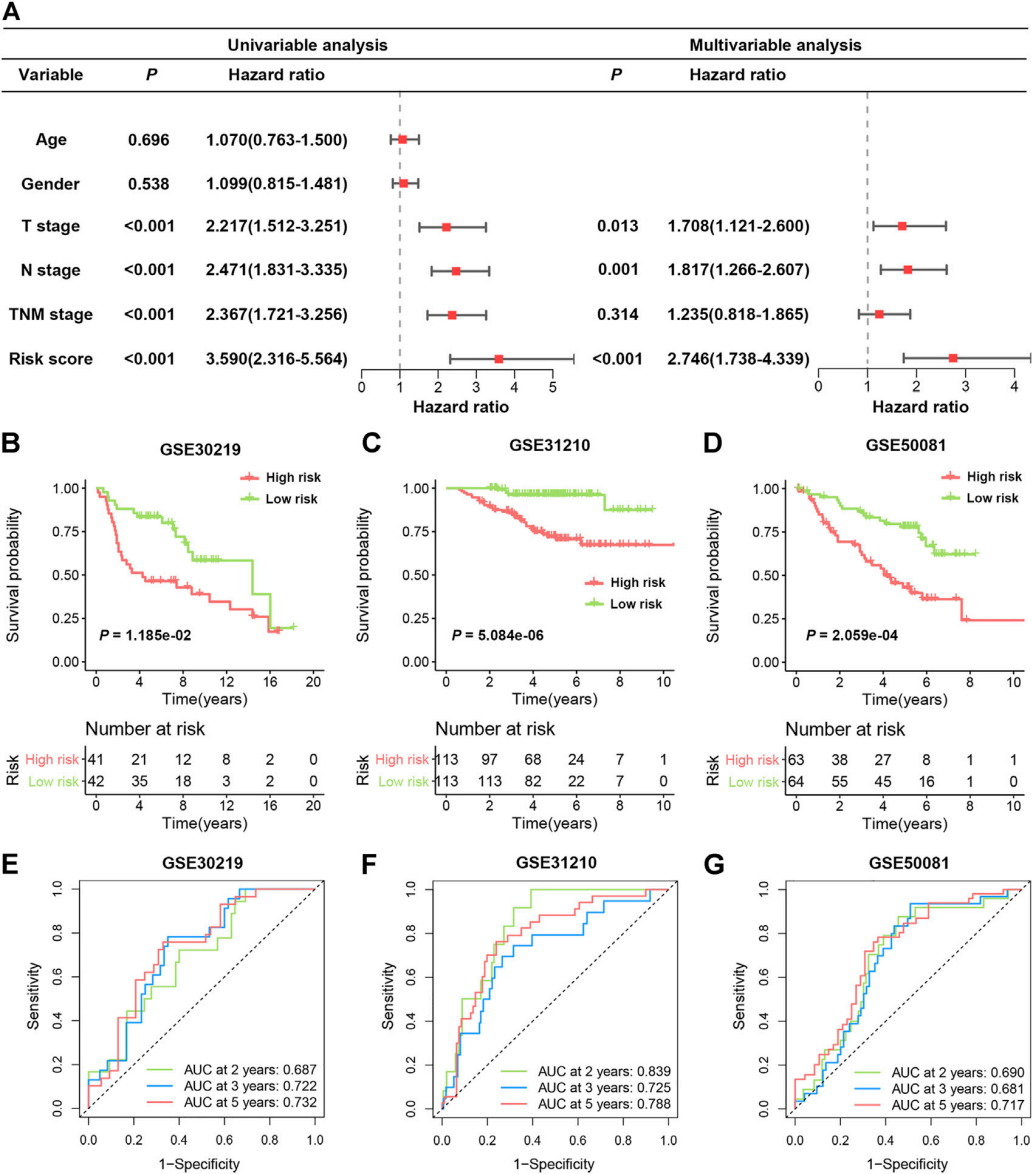
Validation of SRS in independent cohorts. **(A)**. Univariable and multivariable Cox regression analysis of SRS and overall survival in the TCGA cohort. **(B–D)**: Kaplan-Meier curves of OS in high- and low-risk groups in the GSE30219, GSE31210, and GSE50081 datasets. **(E–G)**: ROC curve analyses in the GSE30219, GSE31210, and GSE50081 datasets.

To further verify whether the SRS-based risk score was an independent prognostic factor for LUAD, univariate and multivariate Cox regression analyses of clinicopathological factors in the TCGA cohort were performed. The T stage, N stage, TNM stage and risk score were correlated with OS in univariable analysis. After multivariable adjustment, the risk score remained a significantly independent prognostic factor (HR = 2.746, 95% CI: 1.738–4.339, *p* < 0.001) for patients with LUAD (Figure 4A). We also analysed the correlation between SRS and patients’ clinicopathological parameters, including age, sex, T stage, N stage and TNM stage, in the TCGA cohort. Significantly higher percentages of patients with lymphatic metastasis and late-stage LUAD were identified in the high-risk group (Supplementary Figure S3), indicating that a higher SRS score was related to the malignant progression of LUAD.

### Validation of SRS in Three Independent Cohorts

To validate the predictive function of SRS on OS benefit, three independent data sets from the GEO database were enrolled. As illustrated in Figures 4B–D, patients with high-risk scores exhibited significantly worse OS in all three cohorts, including GSE30219 (HR 2.163, 95% CI 1.188–3.938, *p* = 0.0118), GSE31210 (HR 6.699, 95% CI 3.450–13.01, *p* < 0.0001) and GSE50081 (HR 2.842, 95% CI 1.628–4.962, *p* = 0.0002). The area under the ROC curve (AUC) values in the GSE30219 cohort were 0.687, 0.722, and 0.732 for 2, 3 and 5 years, respectively (Figure 4E). In the GSE31210 cohort, all AUC values were greater than 0.7 (Figure 4F). For the GSE50081 cohort, the AUCs of SRS at 2, 3, and 5 years were 0.690, 0.681, and 0.717, respectively (Figure 4G). Moreover, we also observed that high expression of the five genes in four different cohorts was consistently indicative of poor prognosis for LUAD patients (Supplementary Figure S4). These results confirmed that SRS could serve as a good predictive factor to classify patients with different OS.

### Biological Processes Analysis of SRS

Multicohort evaluation confirmed a robust prognostic value of SRS, which prompted us to further explore the possible mechanism underlying the predictive role of the signature. As shown in Supplementary Figures S5A,B, all five genes were abnormally upregulated in LUAD and many other types of cancer, including colorectal cancer, liver cancer, and brain cancer. We then analysed the relationship between methylation and the expression of the five genes. Significantly lower methylation levels of PTTG1 and TACC3 promoters were found in tumor tissues compared with normal tissues, which may account for the abnormal expression of the signature genes in LUAD (Supplementary Figure S5C).

Regarding the downstream effects, we first extracted the DEGs between subgroups categorized by the risk signature by applying the criteria FDR <0.05 and |log2FC| ≥ 1. In total, 1,164 genes were differentially expressed between the two groups (Supplementary Figure S6A; Supplementary Table S6). Based on these SRS-related DEGs, GO analysis and KEGG analysis were performed. As expected, the results indicated that DEGs were involved in cellular senescence and cell cycle-related biological processes, such as nuclear division and chromosome segregation (Supplementary Figures S6B,C; Supplementary Table S7). In addition, GSEA revealed prominent enrichment in hallmark gene sets, such as MTORC1 signalling, glycolysis, and the unfolded protein response, in the high-risk group compared to the low-risk group in TCGA cohort. Similar trends were also observed in the three validation cohorts (Supplementary Figure S6D). These results suggested a more malignant phenotype in patients with high-risk scores, which may lead to a poor prognosis in LUAD patients.

### SRS Is Associated With Alterations in SASP and Immune Cell Infiltration

**FIGURE 5:**
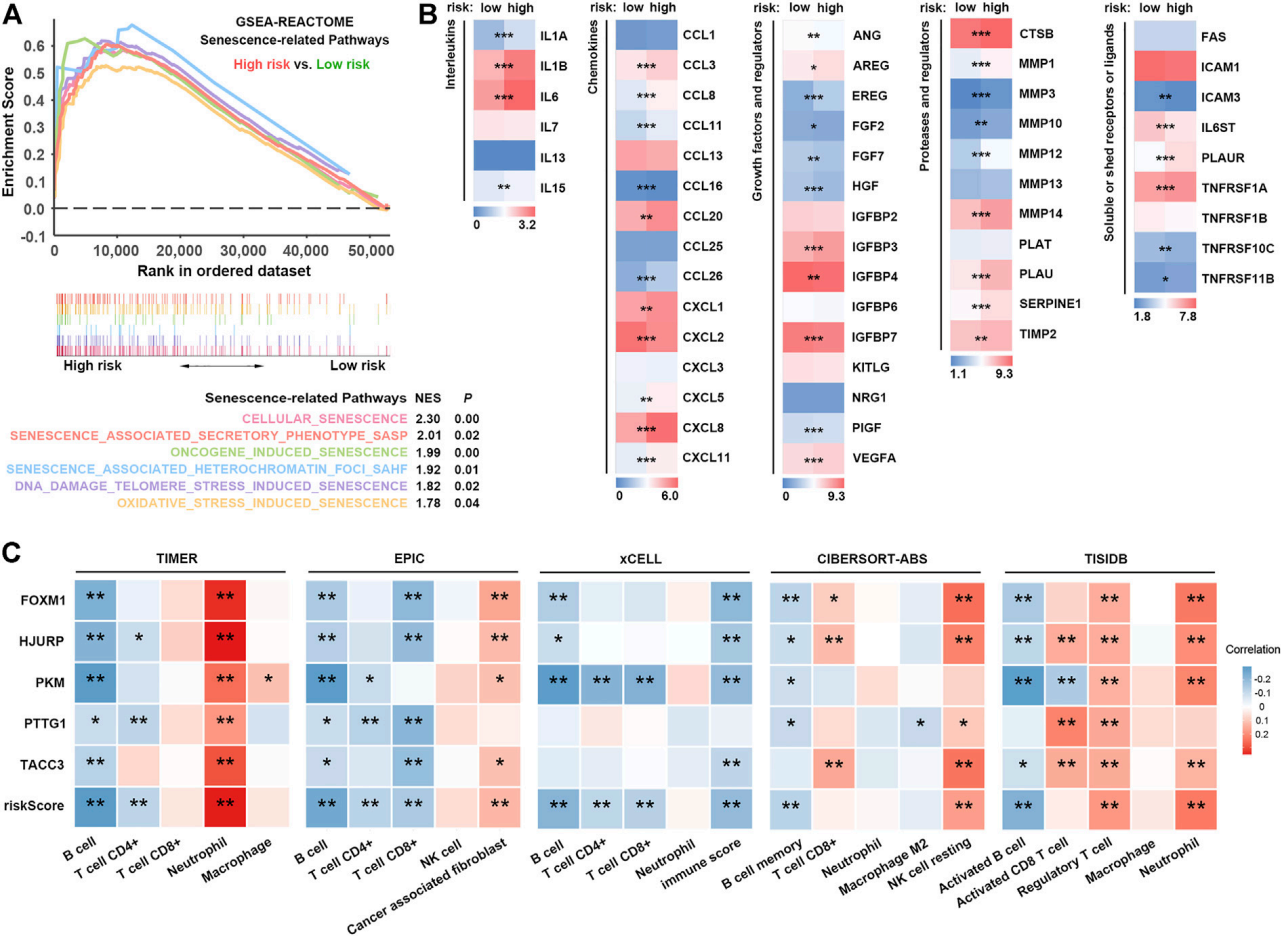
SRS-related SASP and immune cell infiltration. **(A)**. Comparison of signatures related to multiple pathways of cellular senescence between high- and low-risk groups. **(B)**. Expression of different types of SASP factors between high- and low-risk groups. **(C)**. Correlation analysis between risk scores and different immune cells estimated by TIMER, EPIC, xCELL, CIBERSORT-ABS and TISIDB. *, **, and *** represent *P* < 0.05, *P* < 0.01, and *P* < 0.001, respectively.

Cellular senescence occurs when cells are confronted by excessive extracellular or intracellular stress *in vivo* or *in vitro* (Ben-Porath and Weinberg 2004; Ben-Porath and Weinberg 2005). As displayed in Figure 5A, cellular senescence-associated pathways, including oncogene-induced, DNA damage telomere stress-induced, and oxidative stress-induced senescence, were significantly enriched in patients with high SRS scores. Intriguingly, we noticed that the senescence-associated secretory phenotype (SASP) pathway was also prominently enriched. SASP indicates an enormous number of secretory proteins secreted by senescent cells, which may induce changes in the tumor microenvironment, thus promoting tumor recurrence and progression (Acosta, et al., 2008; Coppe, et al., 2008; Green 2008; Kuilman, et al., 2008). Our results revealed overexpression of different types of SASP in the high-risk group (Figure 5B). Interleukins (IL-1A, IL-1B, IL-6, and IL-15), chemokines (CCL3, CCL8, CCL11, CCL20, CCL26, CXCL1, CXCL5, CXCL8, and CXCL11), growth factors and regulators (AREG, EREG, IGFBP3, PIGF, and VEGFA), proteases and regulators (CTSB, MMP1, MMP3, MMP10, MMP12, MMP14, PLAU, SERPINE1, and TIMP2), and soluble or shed receptors or ligands (PLAUR, TNFRSF1A, and TNFRSF11B) were significantly upregulated, further confirming higher levels of the SASP in high-risk patients.

Notably, some upregulated SASPs, including IL-6, CXCL8, and VEGF, possess immunosuppressive properties (Kato, et al., 2018; Lamano, et al., 2019; Liu, et al., 2013; Sharma, et al., 2017). For example, IL-6, secreted by CAFs, regulates immunosuppressive TIL populations in the TIME (Kato, et al., 2018). Thus, we hypothesized that patients with high SRS scores who had higher SASP levels might exhibit an immunosuppressive phenotype via SASP. To characterize the SRS-related immune landscape, RNA-seq-derived infiltrating immune cell populations were estimated by TIMER, EPIC, xCell, CIBERSORT-ABS, and quanTIseq algorithms in TIMER2.0 and TISIDB. We found that patient risk groups stratified by SRS showed distinct immune infiltrate patterns. Correlation analysis showed that the infiltration levels of B cells, CD4^+^ T cells, and CD8^+^ T cells were negatively correlated with the SRS score, whereas a higher SRS score indicated increased abundance of neutrophils, cancer-associated fibroblasts, Tregs, and resting NK cells (Figure 5C; Supplementary Figure S7A). GSEA revealed a significant enrichment of signatures associated with upregulation of TGF-β signalling, whereas no significant difference in IFN-γ signalling was observed in the high SRS score versus the low SRS score group (Supplementary Figure S7B). Taken together, our results implied that a high level of cellular senescence may remodel a suppressive TIME via SASP.

### Impact of SRS and Immune Checkpoints on Clinical Outcome

**FIGURE 6:**
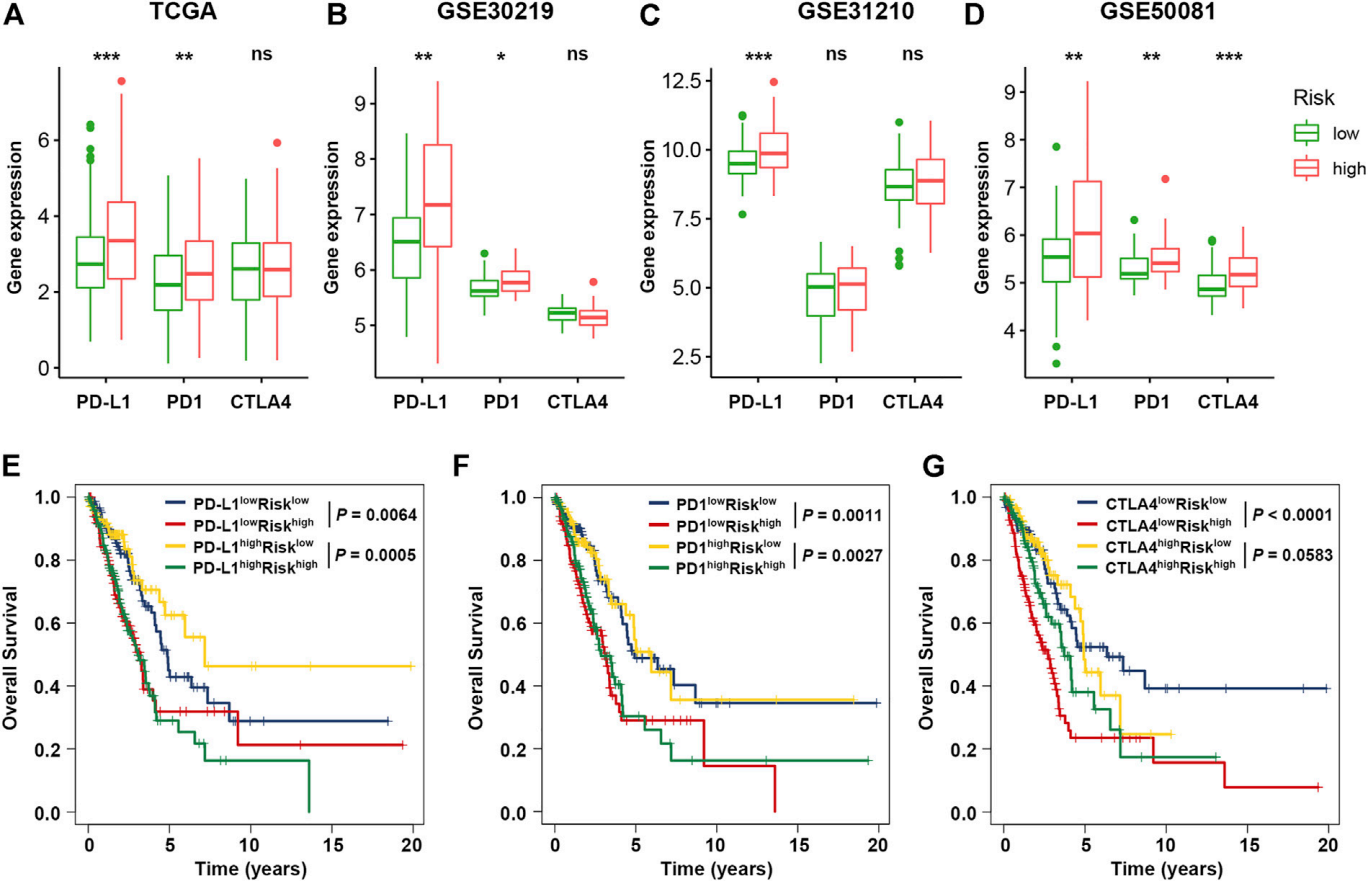
Impact of immune checkpoint genes and SRS on clinical outcome. **(A–D)**. Comparison of the expression levels of immune checkpoint genes (PD-L1, PD1, and CTLA4) between the high- and low-risk groups in the training and validation cohorts. **(E–G)**. Kaplan-Meier survival curves of OS among four patient groups divided by the SRS and PD-L1, PD-1, or CTLA-4. *, **, and *** represent *P* < 0.05, *P* < 0.01, and *P* < 0.001, respectively.

Previous studies have emphasized the importance of immune checkpoint genes in modulating immune infiltration (Juneja, et al., 2017; Kumagai, et al., 2020), and our results also revealed significant relevance between cellular senescence and tumor immunity. Thus, to further investigate the complex crosstalk that occurs among immune infiltration, immune checkpoint genes and SRS, we first compared the expression pattern of immune checkpoint genes between patient groups divided based on the SRS. As shown in Figures 6A–D, patients with high SRS scores tended to express high levels immune checkpoint genes (PD-L1, PD1 and CTLA4) compared with the low SRS group, which was further confirmed in 3 validation cohorts. Other immune checkpoints, such as LAG3 and TIM3, which are also considered exhausted T cell markers, exhibited a trend of overexpression in the high SRS score group in the multicohort, suggesting that SRS has the potential to identify immune dysfunction in LUAD patients (Supplementary Figure S8A).

Next, we considered SRS in combination with immune checkpoint expression to assess whether SRS influences OS in patients with similar immune checkpoint expression. Survival analysis of the four groups stratified by SRS and immune checkpoint gene expression was conducted. As depicted in Figures 6E–G, patients with low PD-L1 and low risk had prolonged OS compared to those with low PD-L1 and high risk (*p* = 0.0064). Among patients with high PD-L1 expression, a lower risk score signified a remarkably better survival (*p* = 0.0005). Similar survival patterns were also observed among the four patient groups stratified by SRS and PD1 or CTLA4 expression in TCGA cohort. Besides, among various immune checkpoint genes, multivariate Cox regression modelling showed that SRS score remained an independently predictor for overall survival (HR = 2.083, 95% CI: 1.533–2.830, *p* < 0.001). We then repeated the same analysis in three validation cohorts. Consistent with TCGA dataset, patients with low SRS scores had significantly better survival than those with high SRS scores despite the fact that similar expression levels of immune checkpoint genes were observed in cohorts GSE50081 (Supplementary Figure S8B) and GSE31210 (Supplementary Figure S8C). However, no significant result was observed in cohort GSE30219 (Supplementary Figure S8D).

In addition to immune checkpoints, TMB is also considered an independent prognostic predictor in various cancer types. We first calculated the TMB of each group and found that patients with higher SRS scores had noticeably increased TMB relative to the low-risk group (Supplementary Figure S9A). Subsequently, the survival distribution of patient groups classified by SRS and TMB level was also compared. As shown in Supplementary Figure S9B, patients with high SRS scores suffered unfavourable OS irrespective of patients’ TMB level.

These results imply that SRS combined with immune checkpoint expression or TMB might serve as a promising predictor of patients’ clinical outcomes.

### Predictive Potential of SRS in Immunotherapy Response

**FIGURE 7:**
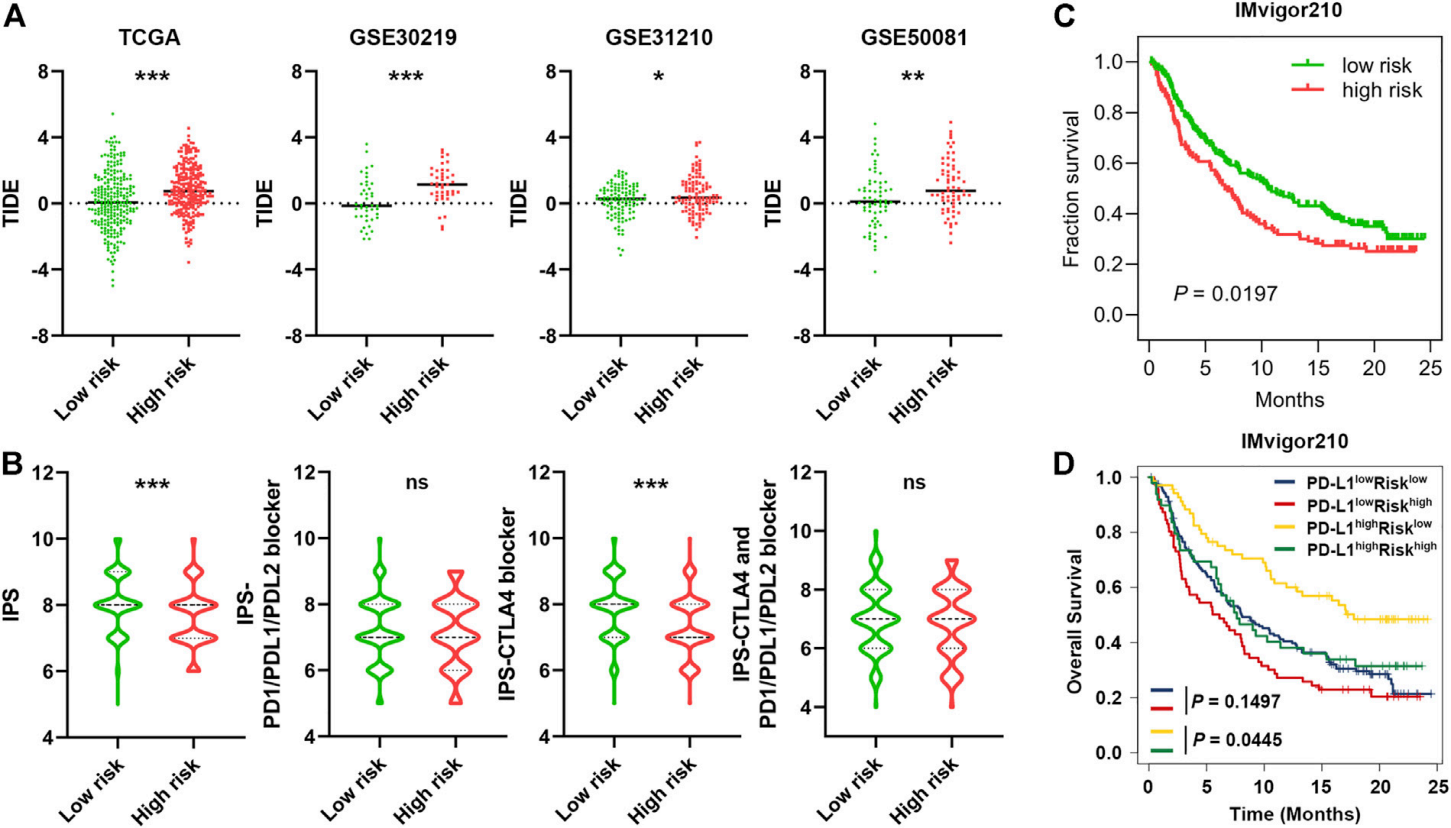
Predictive potential of SRS in immunotherapeutic benefits. **(A)**. The distribution of TIDE scores between patients with a higher SRS score and those with a lower SRS score in four different cohorts as indicated. **(B)**. The distribution of IPS in the high-risk and low-risk groups in the TCGA dataset. **(C)**. Kaplan-Meier curves for high and low SRS score patient groups in the IMvigor210 cohort. **(D)**. Kaplan-Meier curves for four patient groups stratified by SRS and PD-L1 expression. *, **, and *** represent *P* < 0.05, *P* < 0.01, and *P* < 0.001, respectively.

Growing evidence has shown that immune checkpoint inhibitors (ICIs) have improved the survival of NSCLC, but responses vary. Thus, accurate predictive biomarkers are urgently needed (Mok, et al., 2019; Reck, et al., 2016). Given the association between SRS and immune infiltration, we further explored the predictive potential of SRS of ICIs by analysing the correlation of SRS and recognized immunotherapy predictors, including TIDE (Fu, et al., 2020; Jiang, et al., 2018) and IPS(Charoentong, et al., 2017). We discovered that patients in the high-risk group tended to achieve higher TIDE scores in TCGA cohort, and this result was further confirmed in three validation cohorts (Figure 7A). In addition, IPS was significantly increased in the low SRS score group (*p* < 0.001), and patients’ response to anti-CTLA4 treatment was relatively higher in the low-risk group (*p* < 0.001, Figure 7B). These results indicate that patients with low SRS scores may benefit from ICIs.

Considering the immunotherapy response predictive potential of SRS, we next performed Kaplan-Meier survival analysis to investigate the predictive role of immunotherapeutic overall survival using the immunotherapy cohort IMvigor210. As expected, a beneficial trend of low SRS scores in immunotherapeutic OS was observed in the IMvigor210 cohort (HR = 1.368 95% CI 1.036–1.808 *p* = 0.0197, Figure 7C), and the low-risk group also exhibited significantly better OS than the high-risk group among the PD-L1 high (*p* = 0.0445, Figure 7D) or TMB-low population (*p* = 0.0366, Supplementary Figure S9C). Collectively, SRS in combination with TMB or PD-L1 is a promising candidate for predicting the response to ICIs among patients with LUAD and improving therapeutic strategies.

### Validation of Signature Gene Expressions in LUAD Tissues

**FIGURE 8:**
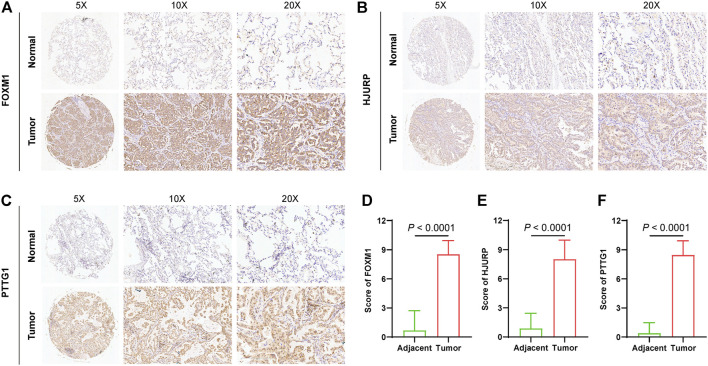
Validation of signature gene expressions in LUAD tissues. Quantification and comparison of protein expression of FOXM1 **(A** and **D)**, HJURP **(B** and **E)**, and PTTG1 **(C** and **F)** by IHC.

To further explore the protein expression of genes that constituted SRS, three genes that exhibited significantly higher expression level in lung cancer in GEPIA2 (Supplementary Figure S5A) were quantified by IHC and compared between in tumor tissues (*n* = 74) and adjacent normal tissues (*n* = 74). As expected, IHC staining revealed that protein expressions of FOXM1, HJURP, and PTTG1 were all significantly elevated in tumors compared with adjacent normal tissues (Figures 8A–F), indicating that SRS genes may play an important in lung cancer progression.

## Discussion

Senescence is a complex biological process with both cell autonomous and paracrine effects that has a significant impact on the microenvironment (Lasry and Ben-Neriah 2015; Hernandez-Segura, et al., 2018). Increasing evidence indicates that senescent cells can be eliminated through a SASP-provoked immune response, which involves both innate and adaptive immunity (Schneider, et al., 2021). Conceivably, the SASP has several positive functions in the short term. However, in the long term, these functions can become detrimental in the immunosuppressive context of cancer to promote tumor development (Lopes-Paciencia, et al., 2019; Coppe, et al., 2010; Basisty, et al., 2020; Birch and Gil 2020). However, how senescent cells interact with tumor immune infiltration and their value in evaluating the immune infiltrate of tumors and clinical outcomes have not been reported, particularly in lung cancer. Thus, modelling lung cancer will be important to decipher whether senescence molecular determinants reshape tumor microenvironments and whether this modification has implications for the prognosis and immunotherapy response of LUAD patients. Importantly, uncovering how cellular senescence influences the TIME can provide a window for discoveries of how we can effectively improve the immunosuppressive milieu by senolytic therapies (van Deursen 2019).

In this study, we analysed the expression patterns, prognostic values, and effects on the TIME of cellular senescence-related genes in LUAD. Using the LASSO method, we constructed a novel survival prediction model (SRS) based on the expression of five senescence features in the TCGA dataset. Furthermore, the SRS was well validated in three different public GEO datasets. We also explored the features of the immune microenvironment, including immune cell distribution and inflammatory activities, in patients with high and low SRS scores. Markedly, we distinguished different SASP affecting TIME remodelling as potential mechanisms underlying immune escape and tumor progression. Additionally, we found that the SRS score was an independent prognostic factor for LUAD patients and was coupled with specific immune checkpoint factors or TMB as predictive biomarkers of ICI response.

This study represents one of the first reports to examine the differential expression analysis of cellular senescence-relevant genes and then identify their prognostic values using TCGA and GEO databases. Markedly, we identified five significantly upregulated genes, including FOXM1, HJURP, PKM, PTTG1, and TACC3, which were also included in the cellular senescence-related signature reported in this study. Interestingly, these five signature genes are reported as negative regulators of cellular senescence in many human cancers and play important roles in tumor development (Ao, et al., 2017; Caporali, et al., 2012; Chen, et al., 2010; Francica, et al., 2016; Kato, et al., 2007; Schmidt, et al., 2010; Tao, et al., 2014). Forkhead box protein M1 (FOXM1) is significantly associated with immunotherapy resistance in lung cancer and patients (Galbo, et al., 2021; Wang, et al., 2014). Holliday junction recognition protein (HJURP), a histone H3 chaperone, affects cell cycle progression, DNA repair and chromosome segregation during mitosis. HJURP is overexpressed in cancers (Wang, et al., 2020) and associated with poor prognosis in NSCLC (Wei, et al., 2019). Pyruvate kinase M (PKM) is a glycolytic enzyme required for tumor proliferation and progression. PKM2 acts as the key factor mediating Th17 cell differentiation (Damasceno, et al., 2020), and silencing PKM2 mRNA could decrease PD-L1 expression and cancer evasion of immune surveillance (Guo, et al., 2019). As an oncogene during spindle formation or chromosome segregation (Bernal, et al., 2002; Li, et al., 2013), pituitary tumor-transforming gene-1 (PTTG1) is an independent poor prognostic factor in NSCLC patients (Wang, et al., 2016) and can elicit an immunogenic response in NSCLC patients (Chiriva-Internati, et al., 2014). Transforming acidic coiled-coil protein 3 (TACC3) is involved in chromosomal alignment, separation, and cytokinesis, which is correlated with p53-mediated apoptosis (Schneider, et al., 2008; Zhang, et al., 2018). Additionally, TACC3 exerts as a prognostic biomarker for prostate cancer (Qie, et al., 2020), osteosarcoma (Matsuda, et al., 2018) and NSCLC (Jiang, et al., 2016), and high TACC3 expression is associated with increased immune cell infiltration and T cell exhaustion (Fan, et al., 2021). These published experimental efforts provide further evidence supporting that SRS has the potential to mirror LUAD prognosis based on immune landscape alterations.

As the role of cellular senescence is largely underexplored in cancer, it is important to gain more extensive insight into the linkage of cancer, senescence and the immune environment. However, to date, the effect of senescence on the tumor immune infiltrate and whether this would impact the response to ICIs have only been poorly studied. By performing a detailed characterization of the tumor immune infiltrate in patients with LUAD, we observed that cellular senescence-related genes could have substantial effects on the composition and distribution of the tumor immune infiltrate. In this study, we found that the SRS score was inversely associated with the infiltration levels of B cells, CD4^+^ T cells and CD8^+^ T cells, whereas neutrophils, CAFs, Tregs and resting NK cells were positively correlated with the SRS score in LUAD. This result suggested that patients with higher SRS score might have an immunosuppressive tumor microenvironment, which prevented immune clearance of tumor cells. GSEA also showed that upregulation of the TGF-β-associated pathway, which has been widely reported as an important factor to restrain antitumor immunity (Mariathasan, et al., 2018; Sheng, et al., 2021), was prominently enriched in the high-risk group. Second, to further explore the mechanisms of immune remodelling by the increasing burden of senescent cells in tumors, we uncovered that alterations of SASP could impact TIME establishment, which ultimately contributes to immune escape and provokes tumor development. The high SRS score group exhibited increases in proinflammatory cytokines, including IL-1b, IL-6, and IL-8; growth factors, such as EGF, VEGF and IGFBP; receptors, such as ICAMs; and proteases, such as MMPs. These factors may modulate immune cell recruitment and have a tumor-promoting effect (Coppe, et al., 2010; Cuollo, et al., 2020; Basisty, et al., 2020; Lau and David 2019). Third, as mentioned above, cellular senescence-related immune remodelling may explain the diminished efficacy of checkpoint blockade. Intriguingly, we noticed that the exhausted T cell markers PD-(L)1, CTLA-4, LAG-3, and TIM3 were aberrantly increased in LUAD specimens with high SRS scores, indicating that T cells become progressively hypofunctional and hyporesponsive with senescence upregulation. This finding may explain the lower response to immunotherapy in older individuals.

Therefore, our findings provide obvious clinical significance. On the one hand, significantly prolonged survival was observed for patients with low SRS scores, suggesting that high-risk patients should receive more frequent clinical surveillance and corresponding measures to prevent disease recurrence and progression. On the other hand, given that only a proportion of patients can derive durable benefits from ICIs, we need more accurate biomarkers with clinical utility. The developed cellular senescence-related signature can be applied not only as a prognostic tool but also as guidance for individualized immunotherapy. Besides, Small molecules targeting FOXM1 (Gormally, et al., 2014; Li, et al., 2020), PKM2 (Ning, et al., 2017) and TACC3(Akbulut, et al., 2020; Polson, et al., 2018) have been developed, and demonstrated promising anticancer capacity *in vitro* and *in vivo* experiments. These findings highlight the potential for using these compounds for future clinical application. Furthermore, we propose that controlling cellular senescence-associated inflammation by targeting specific inflammatory mediators may have a beneficial therapeutic effect in the treatment of cancer. A new group of drugs, named senolytic drugs, including quercetin, navitoclax, and fisetin, have received increased attention, and preclinical clinical data of their potential role in combination with immunotherapy are emerging. Thus, this group of drugs may have vast implications (van Deursen 2019; Campisi, et al., 2019; Kolb, et al., 2021; Prasanna, et al., 2021).

Although our study reports the benefits of immunotherapy and prognosis in LUAD, this study has several limitations. First, the five-gene signature was developed and validated in a public dataset; thus, external validation in multicentre cohorts is needed. Second, it is necessary to perform prospective clinical trials to verify the applicability of our research results in LUAD patients receiving immunotherapy. Third, the regulatory mechanisms by which cellular senescence-related genes reshape the TIME warrant further *in vivo* and *in vitro* investigations. Moreover, further studies are also needed to illustrate how the aged TIME contributes to lung cancer development. Finally, the preliminary interpretation of mechanisms underlying the association between cellular senescence-related genes and worse response to ICIs must be further elucidated using basic experiments.

In conclusion, our study identified and validated a cellular senescence-related signature that is based on five cellular senescence-related genes as an indicator of immune cell infiltration in the TIME and had independent prognostic significance for patients with LUAD. Importantly, the SRS was significantly associated with the immune cell infiltration levels of LUAD patients and involved in the regulation of the LUAD immune microenvironment by SASP. Finally, we characterized the complex interplay between the SRS score and immune checkpoint genes in patient outcomes and suggested the potential of the SRS score coupled with specific immune checkpoint factors as predictive biomarkers of ICI response to enable a more precise selection of patients who will benefit from checkpoint inhibitor immunotherapy. Therefore, identifying cellular senescence-related genes affecting tumor immune responses and further studying their regulatory mechanisms might assist risk stratification and provide promising targets for improving the response of LUAD to immunotherapy.

## Data Availability

The original contributions presented in the study are included in the article/Supplementary Material, further inquiries can be directed to the corresponding authors.
