# Supplementary material for: Comprehensive Analysis Uncovers Prognostic and Immunogenic Characteristics of Cellular Senescence for Lung Adenocarcinoma
Source: Front Cell Dev Biol. 2021 Nov 16;9:780461. doi: 10.3389/fcell.2021.780461 (PMC8636167; doi:10.3389/fcell.2021.780461)
Supplement: Supplementary file 1 [file DataSheet1.pdf]

Figure S1

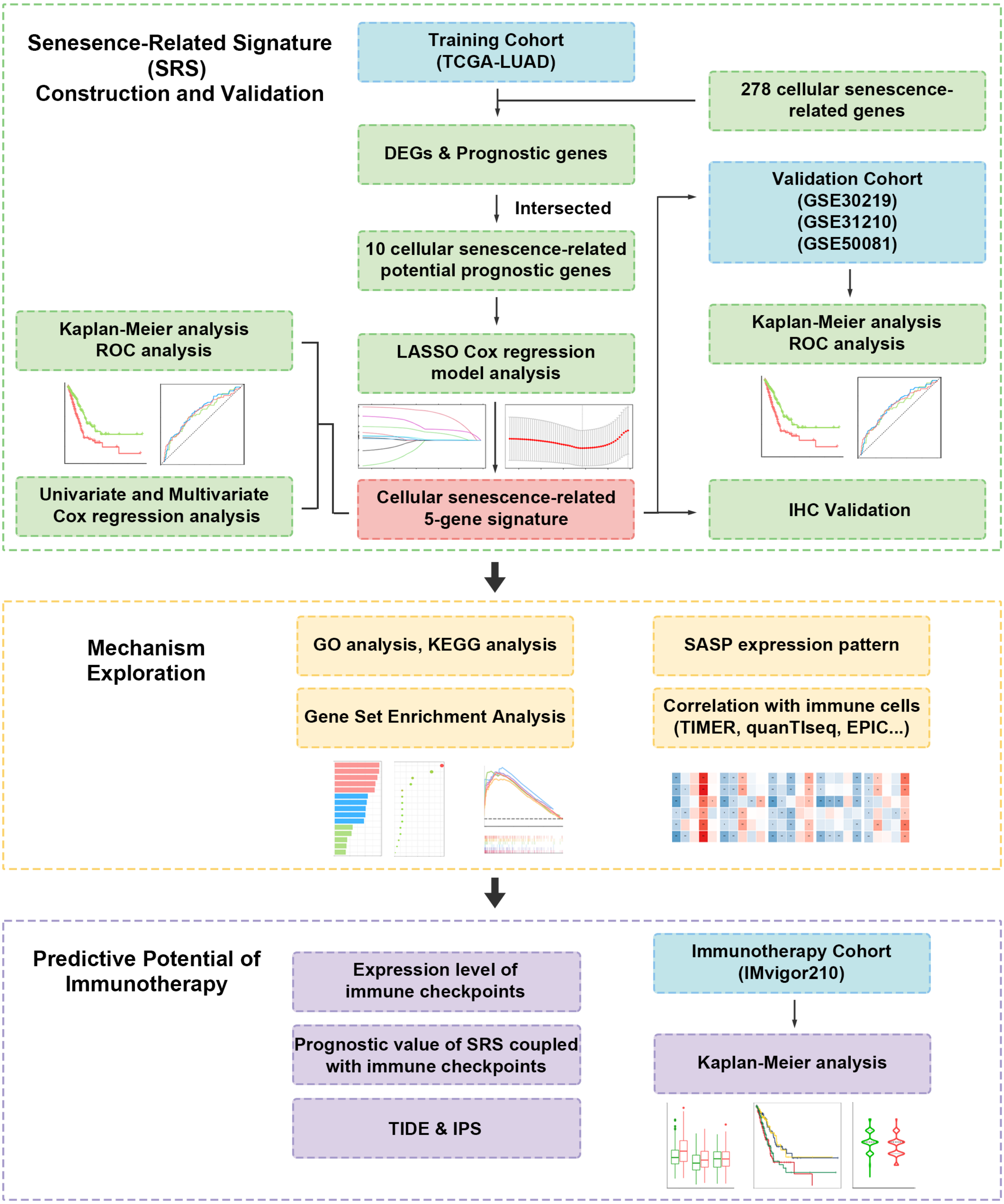

Figure S1. The flow diagram of this study.

Figure S2

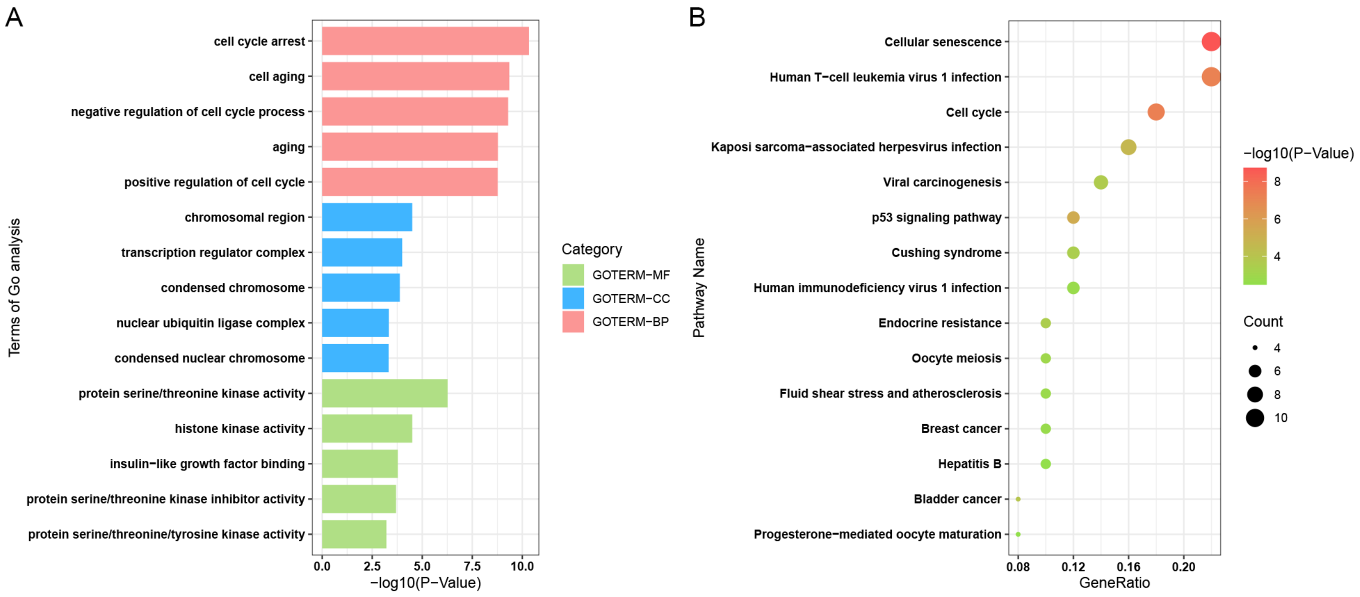

Figure S2. GO enrichment analysis (A) and KEGG pathway enrichment analysis (B) of the differentially expressed cellular senescence-related genes.

Figure S3

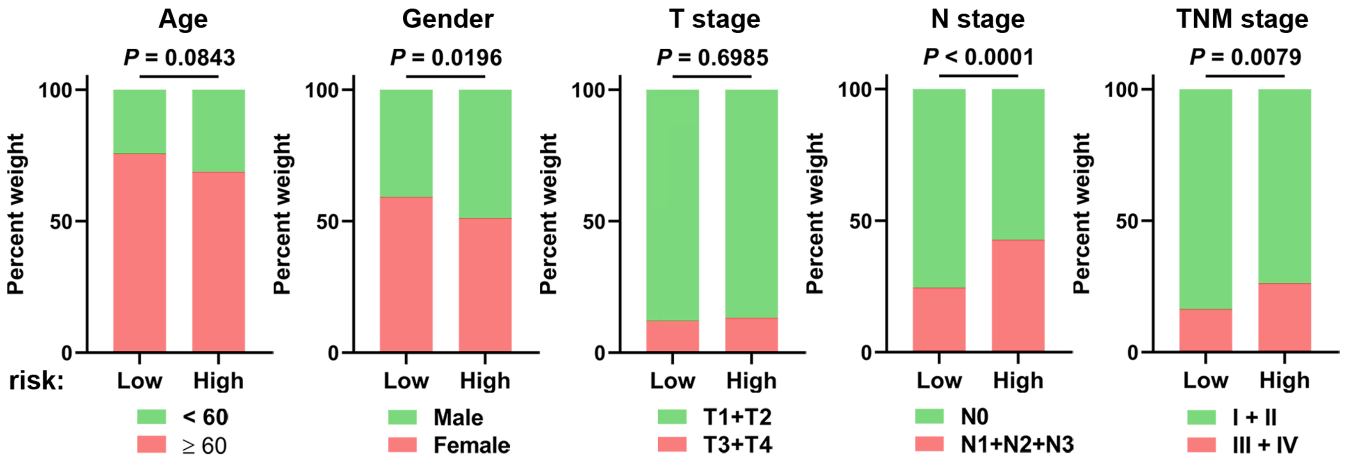

Figure S3. The correlation between SRS and patients' clinicopathological parameters, including age, sex, T stage, N stage and TNM stage.

Figure S4

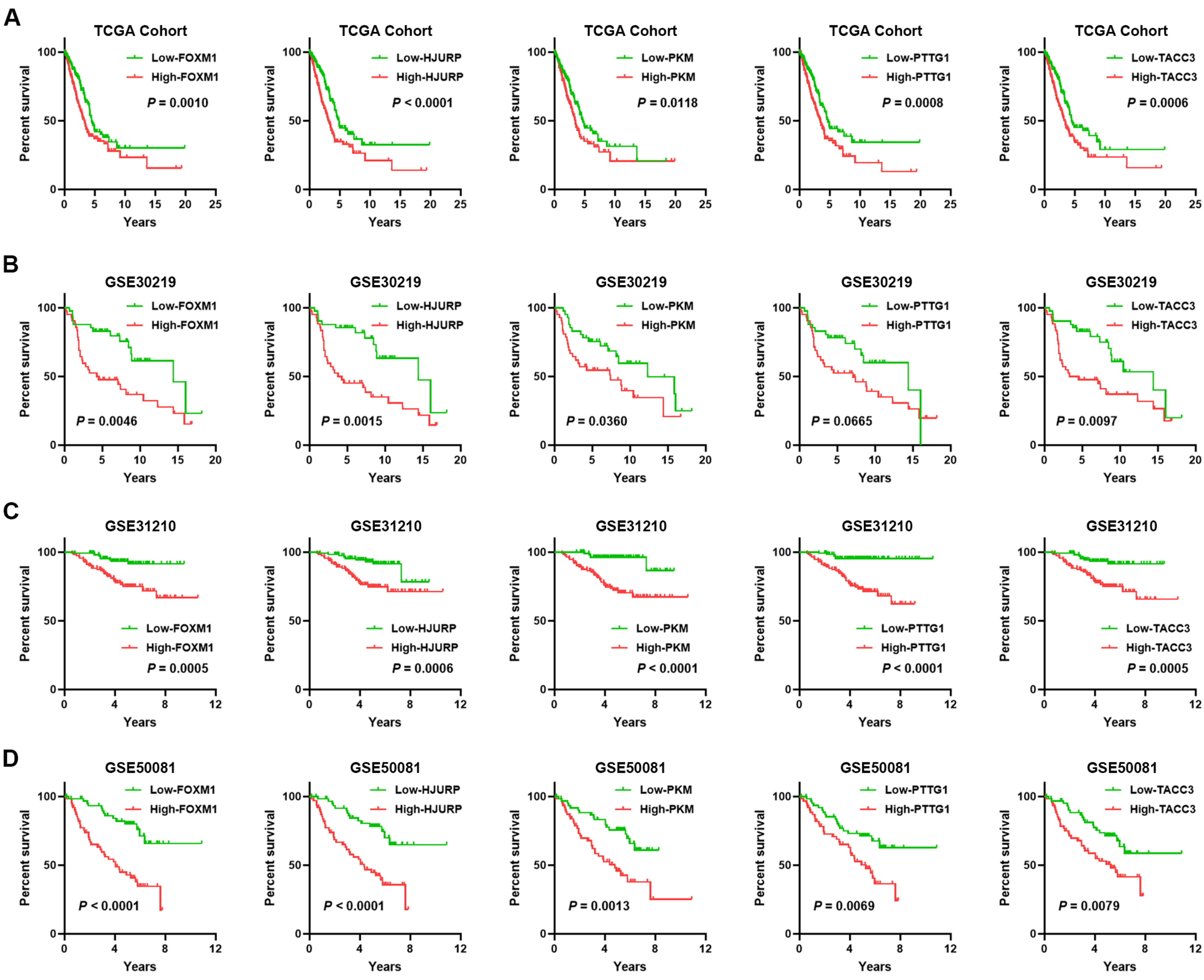

Figure S4. Kaplan-Meier curves for patients with high and low expression of FOXM1, HJURP, PKM, PTTG1 and TACC3 in both the training (A) and validation (B-D) cohorts.

Figure S5

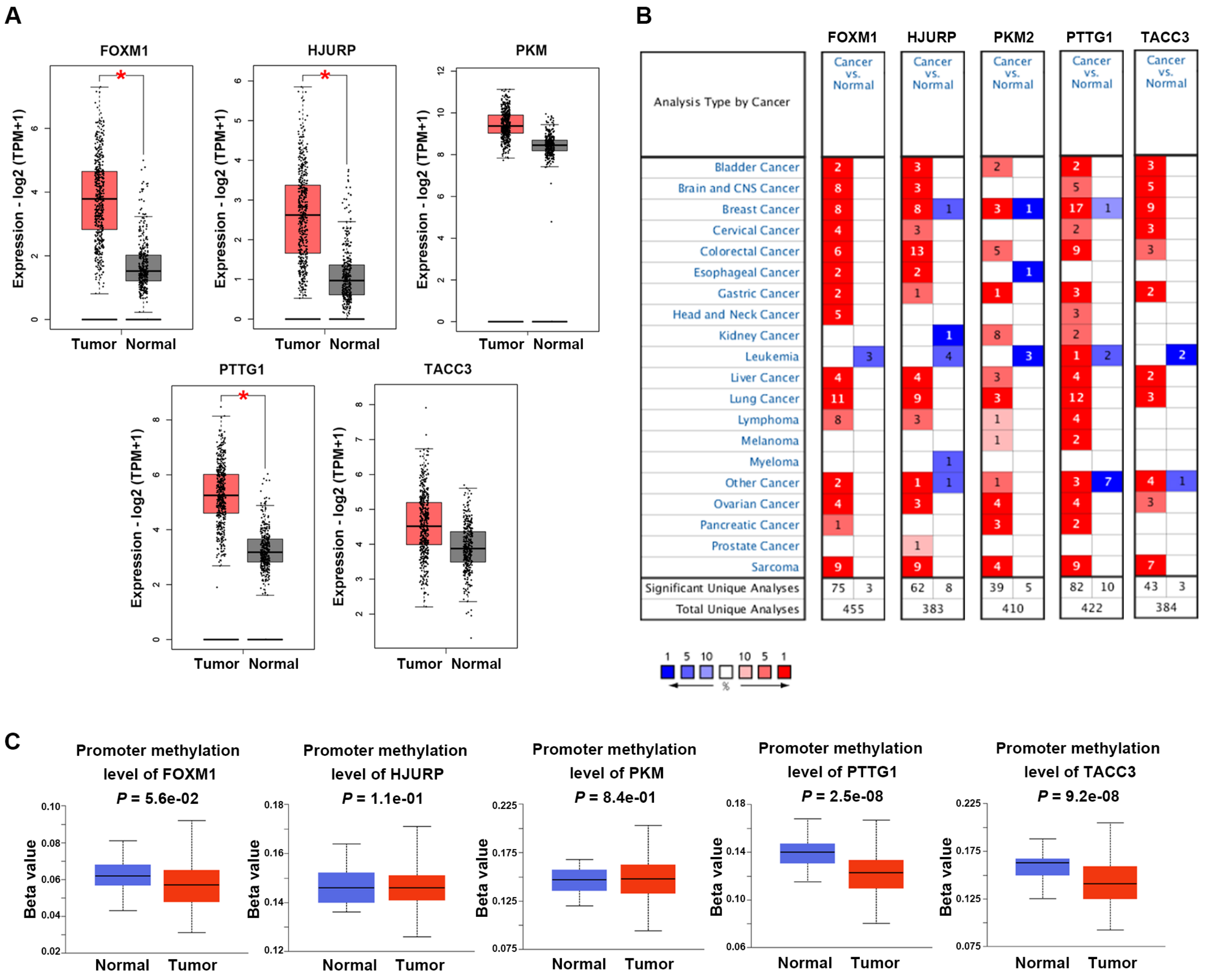

Figure S5.

A. Analysis of FOXM1, HJURP, PKM, PTTG1 and TACC3 expression in GEPIA2, * represents P < 0.05.

B. Pancancer analysis of FOXM1, HJURP, PKM, PTTG1 and TACC3 expression in Oncomine.

C. Promoter methylation levels of the five genes between normal and tumour tissues were evaluated using UALCAN.

Figure S6

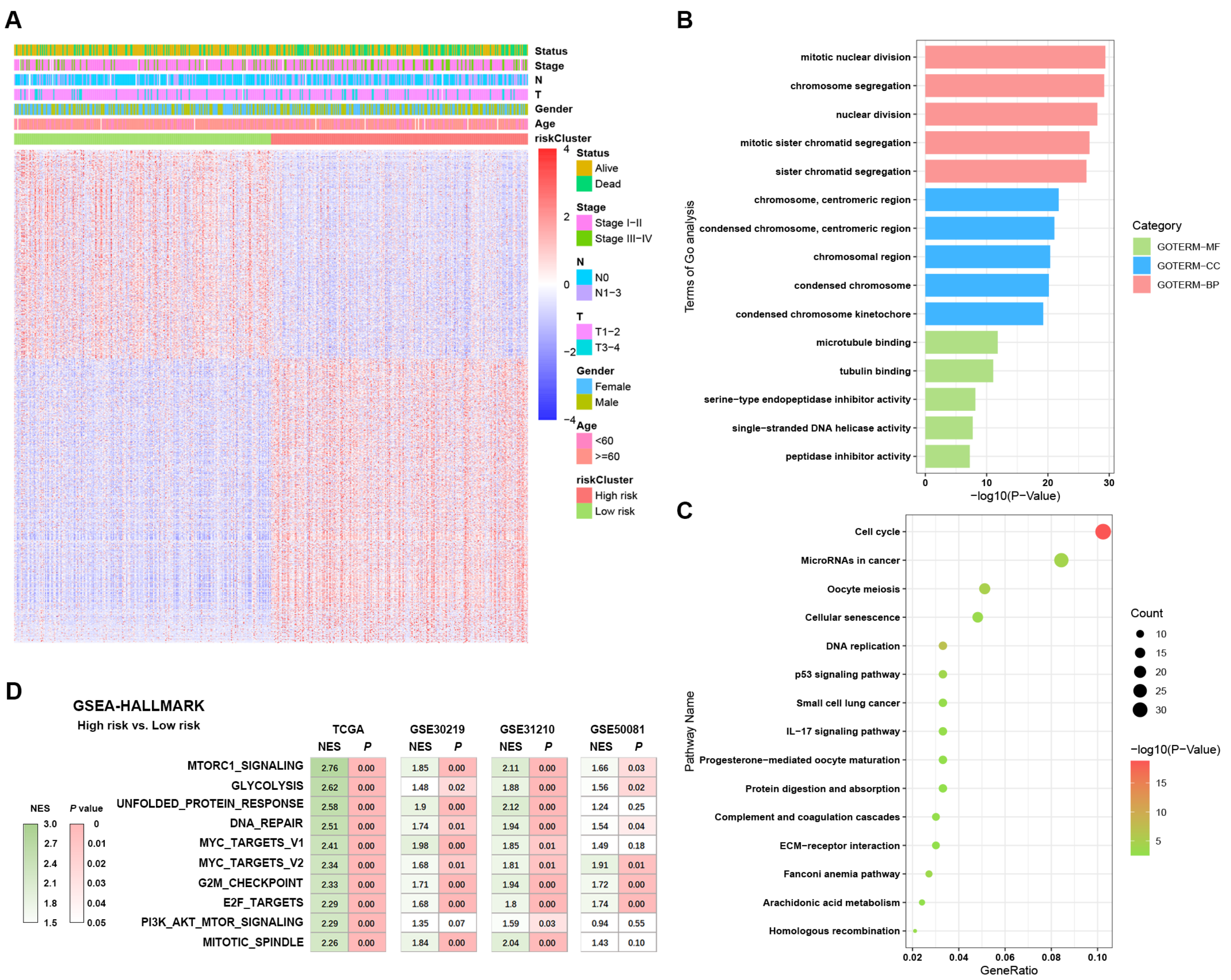

Figure S6.

A. Heatmap of significantly differentially expressed genes between the high- and low-risk groups.

B. GO enrichment analysis of the DEGs.

C. KEGG pathway enrichment analysis of the DEGs.

D. GSEA of hallmark gene sets compared between high-risk and low-risk groups.

Figure S7

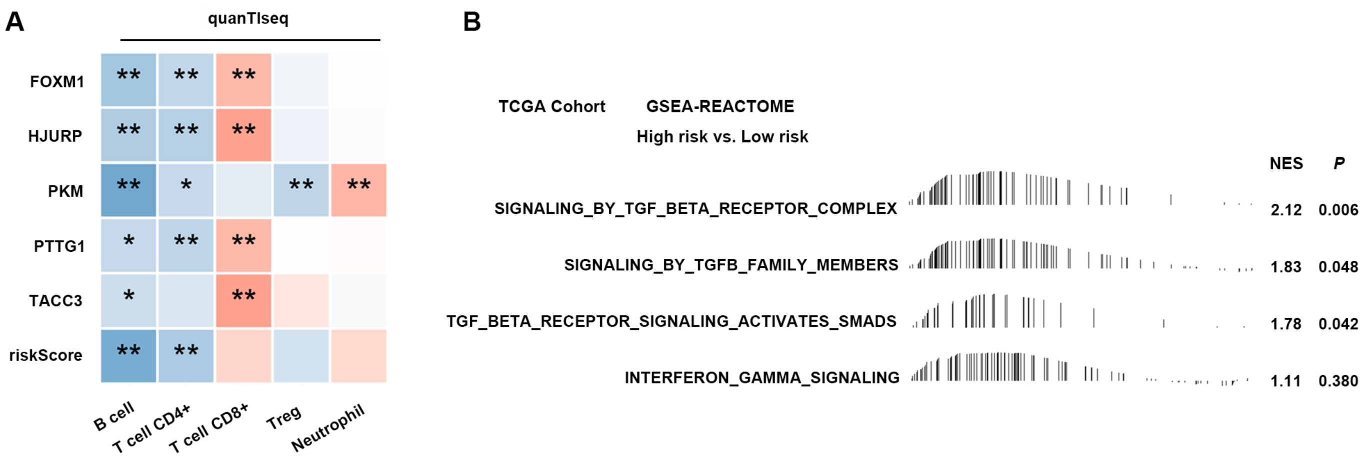

Figure S7.

A. Correlation analysis between risk scores and different immune cells estimated by quanTIseq.

B. GSEA of IFNγ- and TGFβ-related gene signature comparisons between high- and low-risk groups.

* and ** represent $P < 0.05$ and $P < 0.01$, respectively.

Figure S8

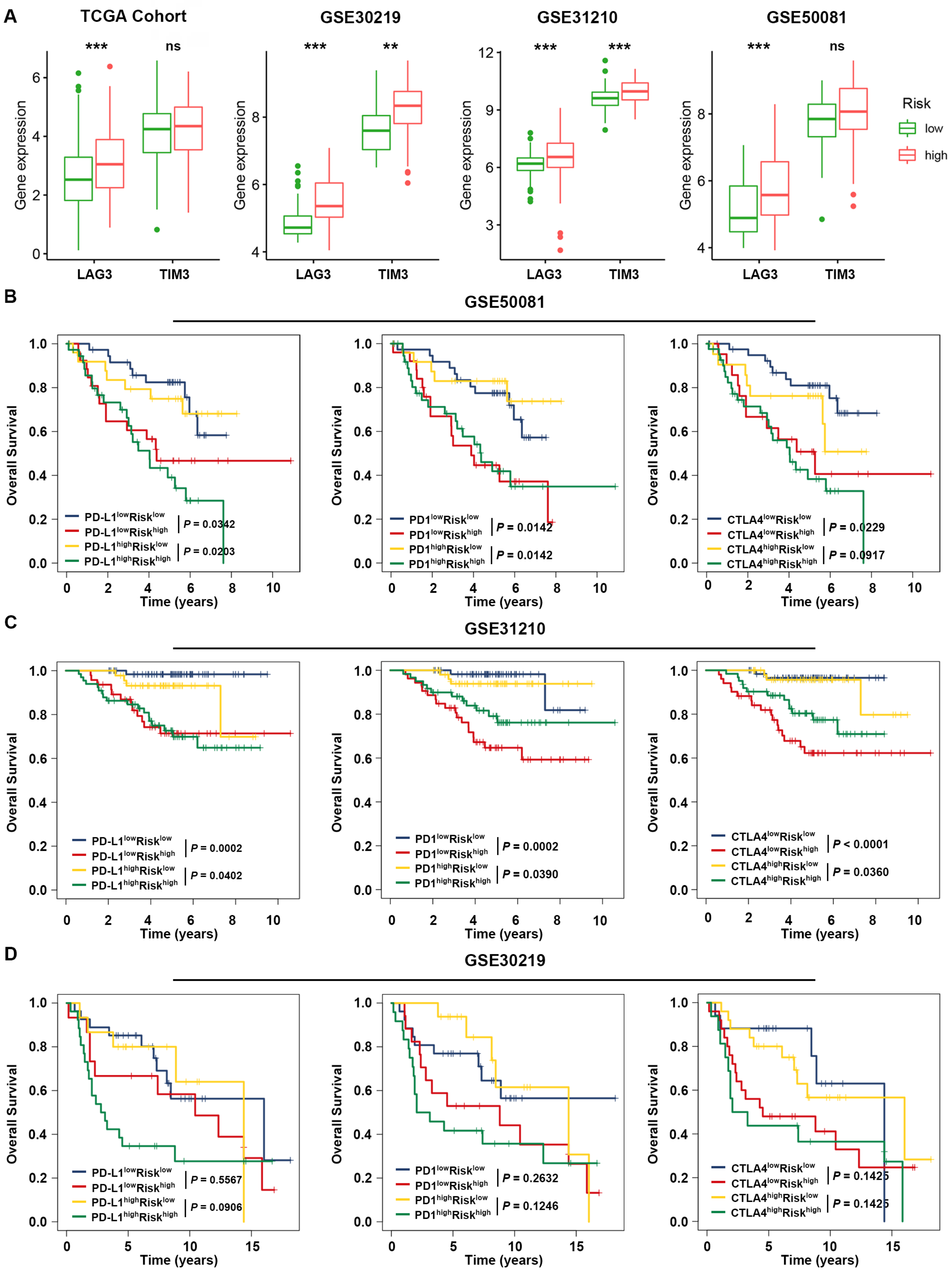

Figure S8.

A. Comparison of the expression level of T cell exhausted markers (LAG3 and TIM3) between high- and low-risk groups in the training cohort and validation cohorts.

B-D. Kaplan-Meier survival curves of OS among four patient groups divided by the SRS and immune checkpoints (PD-L1, PD-1 and CTLA4) in validation cohorts.

** and *** represent P < 0.01 and P < 0.001, respectively.

Figure S9

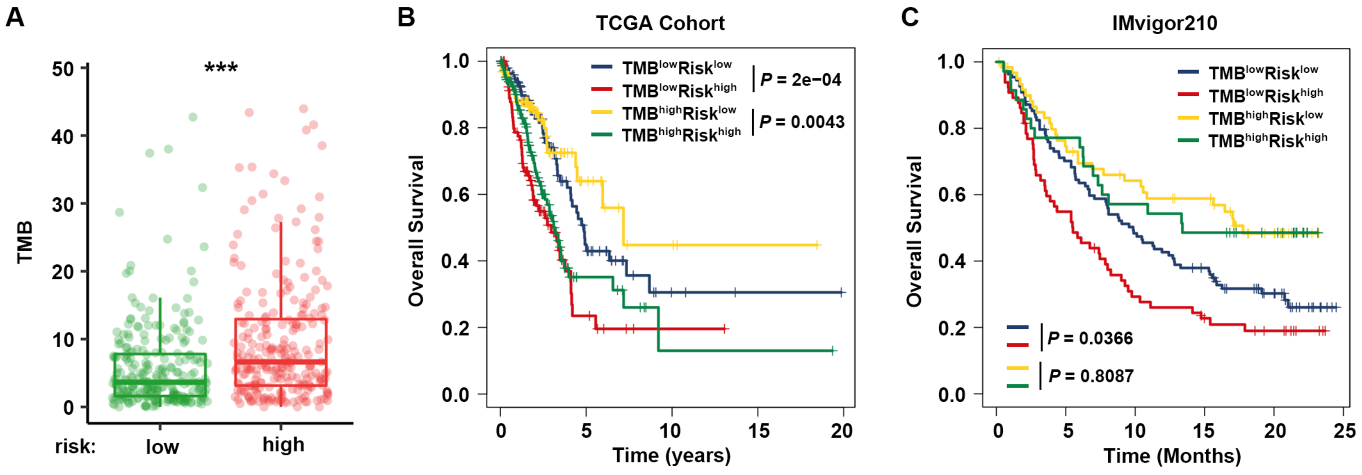

Figure S9.

A. Comparison of TMB in the high- and low-risk groups, *** represents P < 0.001.

B-C. Kaplan-Meier curves for four patient groups stratified by SRS and TMB in the TCGA cohort and IMvigor210 cohort.
